# *Ae*DES: a next-generation monitoring and forecasting system for environmental suitability of *Aedes*-borne disease transmission

**DOI:** 10.1038/s41598-020-69625-4

**Published:** 2020-07-28

**Authors:** Á. G. Muñoz, X. Chourio, Ana Rivière-Cinnamond, M. A. Diuk-Wasser, P. A. Kache, E. A. Mordecai, L. Harrington, M. C. Thomson

**Affiliations:** 1 International Research Institute for Climate and Society (IRI), The Earth Institute at Columbia University, Palisades, New York, NY 10964 USA; 2 Pan-American Health Organization (PAHO), World Health Organization (WHO), Washington, DC USA; 3 Department of Ecology, Evolution and Environmental Biology, Columbia University, New York, NY 10027 USA; 4 Biology Department, Stanford University, Stanford, CA 94305 USA; 5 Department of Entomology, Cornell University, Ithaca, NY 14853 USA; 6 Wellcome Trust, London, NW1 2BE UK

**Keywords:** Infectious diseases, Environmental sciences, Climate sciences

## Abstract

*Aedes*-borne diseases, such as dengue and chikungunya, are responsible for more than 50 million infections worldwide every year, with an overall increase of 30-fold in the last 50 years, mainly due to city population growth, more frequent travels and ecological changes. In the United States of America, the vast majority of *Aedes*-borne infections are imported from endemic regions by travelers, who can become new sources of mosquito infection upon their return home if the exposed population is susceptible to the disease, and if suitable environmental conditions for the mosquitoes and the virus are present. Since the susceptibility of the human population can be determined via periodic monitoring campaigns, the environmental suitability for the presence of mosquitoes and viruses becomes one of the most important pieces of information for decision makers in the health sector. We present a next-generation monitoring and forecasting system for $\underline{\textit{Ae}}{} \textit{des}$-borne diseases’ environmental suitability (*Ae*DES) of transmission in the conterminous United States and transboundary regions, using calibrated ento-epidemiological models, climate models and temperature observations. After analyzing the seasonal predictive skill of *Ae*DES, we briefly consider the recent Zika epidemic, and the compound effects of the current Central American dengue outbreak happening during the SARS-CoV-2 pandemic, to illustrate how a combination of tailored deterministic and probabilistic forecasts can inform key prevention and control strategies .

## Introduction

Human society is more and more interconnected every year by communication technologies, travel and supply chains. As a consequence, increasing movement of humans, animals, pathogens, vectors, goods, and capital across borders creates both risks and opportunities^1^. Like climate, epidemics do not mind political borders, and can impact social stability and human health. In the last couple of decades, the appearance of a variety of new epidemics, such as the SARS coronavirus in 2003, the avian influenza (H1N1) in 2009, the Ebola virus in western Africa (2014–2016), the Zika virus in the Americas (2015–2016), and the novel coronavirus (SARS-CoV-2) identified in late December 2019 in Wuhan (China) and still ongoing, amongst others, demonstrates how fast emerging infectious diseases can spread, sometimes causing damage at national or regional scale, and other times—like the present SARS-CoV-2 pandemic—impacting the entire world^2^.

Multiple infectious diseases are climate-sensitive, with climate acting as a key driver of spatio-temporal patterns of infections, related to seasonal, year-to-year, and longer-term shifts in populations at risk^3^. Climate impacts both pathogens and vectors. Arboviruses of global public health importance, including Zika, dengue, yellow fever, chikungunya, and Rift Valley Fever, have mosquitoes as part of their epidemiological cycles.

Some *Aedes*-borne diseases have experienced an overall increase of 30-fold in the last 50 years, causing more than 50 million infections worldwide every year^4^. In the United States of America, the vast majority of *Aedes*-borne infections are imported from endemic and often neighboring regions—like the Caribbean, Central and South America—by travelers who become potential new sources of transmission. Autochthonous transmission in the continental USA has been already observed for chikungunya virus (2013) and Zika (2017), and risks are likely to increase with anthropogenic global warming as temperatures become more suitable for transmission in temperate regions.

For autochthonous transmission to occur, the population needs to be susceptible to the disease, but there must also be suitable environmental conditions (e.g., suitable temperatures) for both the mosquitoes and the virus. Environmental suitability for presence of mosquitoes and virus transmission is one of the most important pieces of information for decision makers in the health sector. Moreover, the transmission rates or the number of cases are generally more difficult to forecast than environmental suitability, due to their link to a larger number of (often entangled and more complex) predictors, involving human behavior and socio-economic conditions.

A generalized approach to modeling *Aedes*-borne pathogens is needed because multiple *Aedes* species can serve as vectors of dengue, Zika, and chikungunya. Although *Aedes aegypti* is the most common vector, *Aedes albopictus* (otherwise known as the Asian tiger mosquito) has been identified as another important vector because of its vector competence for several arboviruses and recent rapid spread^5^. Both vectors pose a potent threat to global health security given their ability to transmit a wide variety of emerging and re-emerging arboviruses for which there are no vaccines. *Aedes aegypti* and *Aedes albopictus* are ubiquitous in large regions of the Americas and the Caribbean.

Historical, current, and forecast climate information can be combined with disease models to improve climate-sensitive health planning and targeting of resources. A typical approach for infectious disease modelling has been to explore different interventions scenarios in order to inform priorities for decision makers^6^. Nonetheless, there is increasing interest in using models for real-time forecasting and climate-and-health services^7–9^, with still important gaps in the operational readiness of several forecasting systems proposed in the literature^10^. Stochastic models are frequently used in climate and disease modeling to build probabilistic forecasts^11^, as they provide a more reliable assessment of the range of likely outcomes. However, probabilistic models are sometimes harder for decision makers to interpret, and tend to be rejected in favour of simpler, deterministic, but over-confident, models. An approach that takes full advantage of both deterministic and probabilistic forecasts is presented and discussed in the following pages.

Although the historical (average) seasonal behavior and similar statistics of these diseases are useful^12^, we consider it not enough for decision-making, as inter-annual variability (e.g., related to El Niño-Southern Oscillation) tends to play an important role in the actual observed variations of *Aedes*-borne diseases, enhancing or reducing the associated risk^8,13–16^. Hence, a formal forecast system and its associated skill assessment is required and, to the best of our knowledge, is still nonexistent for the continental United States and its transboundary regions.

Here, we describe the *Ae*DES ($\underline{\textit{Ae}des}$-borne diseases’ environmental suitability) system, a new pattern-based calibrated, multi-model ensemble of climate-driven *Aedes*-borne disease models for North America, Central America, northern South America and the Caribbean, based on prior work undertaken in collaboration with the Pan American Health Organization (PAHO)/World Health Organization (WHO)^8,16,17^. We built the *Ae*DES system using the same general approach for both its monitoring and forecasting sub-systems, which in addition to supporting surveillance operations, simplifies the forecast verification process. We discuss the use of *Ae*DES to inform concrete prevention and control strategies, using the recent Zika epidemic as an example.

**Figure 1:**
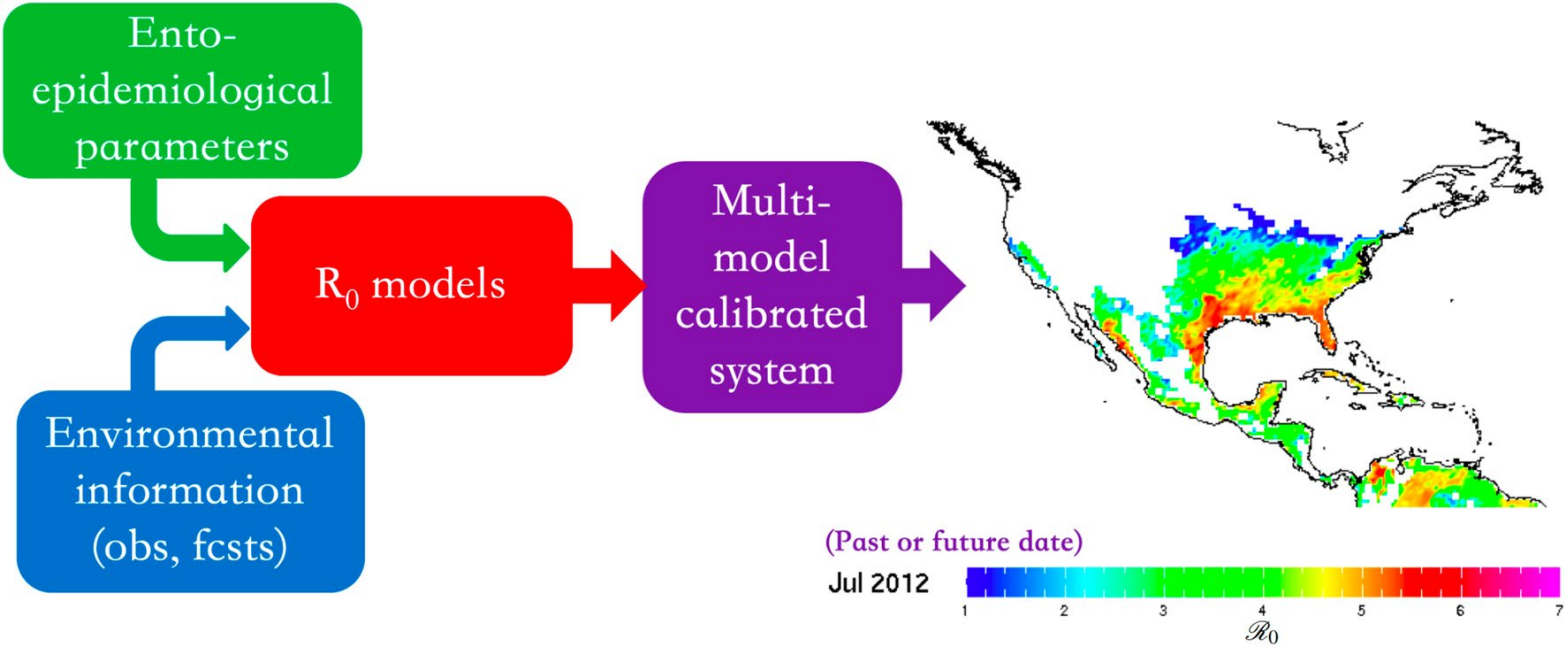
Monitoring and forecast system schematics. Ento-epidemiological and environmental information (obs: climate observations, fcsts: climate forecasts) are used to force four ${\mathscr {R}}_0$ models. Each model is independently calibrated using a pattern-based post-processing approach before being combined.

## Results

*Ae*DES uses multiple ento-epidemiological models to produce estimations of environmental suitability for transmission of *Aedes*-borne diseases, quantified via the basic reproduction number, ${\mathscr {R}}_0$ (red box in Fig. 1). We used the basic reproduction number to assess the environmental suitability of transmission of *Aedes*-borne diseases because (a) it is one of the operational outbreak indices used by WHO and several other decision-making institutes and health practitioners^18,19^, and (b) it has an intuitive interpretation in terms of the number of secondary human cases one case generates on average over the course of its infectious period (assuming a completely susceptible population)^20^; hence, values smaller than one indicate that environmental conditions are not suitable for disease propagation, and the larger the value of ${\mathscr {R}}_0$, the more suitable the conditions are.

Formally, ${\mathscr {R}}_0$ is an environmental suitability (or potential) index for transmission, and not a proper transmission or risk index itself; the latter depends on more complex interactions and the definition of the involved vulnerability, which is in part a function of the susceptibility, exposure and adaptive capacity of the population to the pathogen. In addition, the formalism leading to the definition of ${\mathscr {R}}_0$ involves a series of assumptions (e.g., constant population size, constant transmission and removal rates, no demography and well-mixed population) that makes it just an approximation to the observed behaviour of disease transmission. Thus, as only a measure of environmental suitability, ${\mathscr {R}}_0$ is a necessary but not sufficient condition for transmission. Only if additional information is available for a particular location about the presence of the vector, the circulation of the virus and the vulnerability of the population, can the decision maker interpret ${\mathscr {R}}_0$ values in the *Ae*DES system as a proxy for risk of transmission.

${\mathscr {R}}_0$ works both as a suitability monitoring index—when computed using observed variables, or when estimated by an authoritative organization such as PAHO or the Center for Disease Control (CDC)—and as a forecast index when using actual climate forecasts of the variables required for its computation.

${\mathscr {R}}_0$ models require a set of ento-epidemiological parameters (green box, in Fig. 1) and environmental information, either actual observations if we focus on the monitoring sub-system, or forecasts if we focus on the prediction sub-system (see blue box in Fig. 1). Typically, ${\mathscr {R}}_0$ models require near-surface (2-m) temperatures, but other environmental variables are also involved, like rainfall or even humidity. Here, we use four ${\mathscr {R}}_0$ models already described in the literature, and that ultimately depend only on surface temperature: the Caminade et al.^21^, Wesolowski et al.^22^, Liu-Helmersson et al.^23^ and Mordecai et al.^24^ models. For details see the "Methods"section. We use multiple ${\mathscr {R}}_0$ models to be able to better assess uncertainties, and we calibrate each of the models independently before creating the multi-model ensemble to minimize systematic errors.

**Figure 2:**
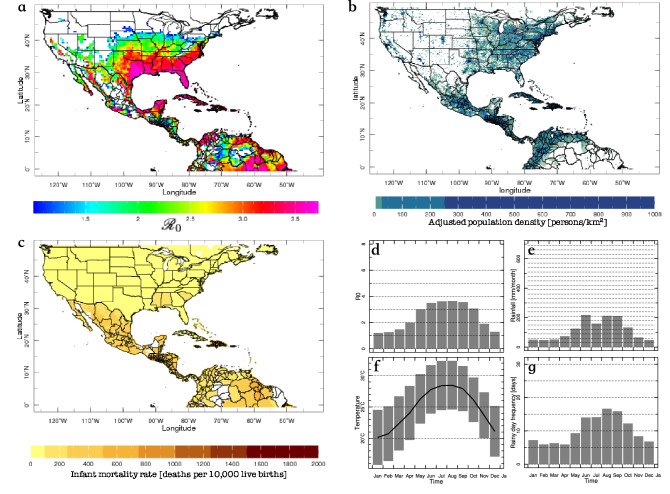
Example of fields available in the *AeDES* monitor system: (**a**) basic reproduction number for July 2019 (only locations with ${\mathscr {R}}_0>1$, suitable for transmission, are plotted); (**b**) population density (persons per square kilometer; estimated for 2020); (**c**) infant mortality (infant deaths per 10,000 live births); and seasonality of (**d**) ${\mathscr {R}}_0$, (e) accumulated rainfall, (**f**) maximum, average and minimum temperature and (**g**) frequency of rainy days for Miami, FL (any other location in the map can be plotted). Only values corresponding to suitable conditions for transmission (${\mathscr {R}}_0>1$) are plotted. In the *AeDES* monitoring sub-system, the user is able to evaluate information on population demographics, social vulnerability, and climate in conjunction with the ${\mathscr {R}}_0$ value to assess potential risk of transmission.

### Monitoring sub-system

The *Ae*DES monitoring sub-system offers maps showing the spatial distribution of environmental suitability over the region of study for the 1948-present period, at a monthly timescale. It also includes additional information to provide context to the user (Fig. 2). These fields were included in the *Ae*DES Maproom (https://aedes.iri.columbia.edu) after consultation with decision makers at PAHO.

To produce the environmental suitability maps (e.g., Fig. 2a), each one of the four ${\mathscr {R}}_0$ models was run from 1948 to present, forced by GHCN-CAMS temperature data^25^ ($\sim$ 56 km resolution) and ento-epidemiological parameters (see “Methods” section), and then combined. The monitoring sub-system is automatically updated in the *Ae*DES Maproom around the 8th day of each month. These maps are useful to know the recent behavior of environmental suitability, or to conduct comparisons with respect to particular years. Trends and variability analysis, or the extension of the northern border of environmental suitability can also be easily computed with this new dataset. As discussed earlier, values of ${\mathscr {R}}_0>1$ in these maps should not be identified as actual transmission of pathogens in those locations, unless there is confirmed evidence of the presence of the vector and circulation of the pathogens, as well as information about the susceptibility and even the lifestyle of the human population^15,26^. The same applies to the forecast maps described in the next section.

The additional information, such as population density (Fig. 2b) and social vulnerability (Fig. 2c), is offered to the user to assess potential risk of transmission and is not part of the models themselves. Once a location is selected, the seasonality of ${\mathscr {R}}_0$, accumulated rainfall, minimum, average and maximum temperatures, and frequency of rainy days (Fig. 2d–g) is provided. Our team is working on adding fields such as human mobility and connectivity, which local experts in the northeast of the US have suggested as also useful to analyze potential disease transmission^27^, consistent with previous research^28–30^.

### Forecast sub-system calibration and evaluation

As indicated earlier, the forecast sub-system employs the output of state-of-the-art climate models and the same ${\mathscr {R}}_0$ models used by the monitoring system. Models, nonetheless, require statistical post-processing to help correct for biases with respect to the monitored ${\mathscr {R}}_0$ values. Following Muñoz et al.^8^, a pattern-based Model Output Statistics (MOS) approach using principal component regression (PCR) was applied to the raw ${\mathscr {R}}_0$ models output. Since the ${\mathscr {R}}_0$ models are the same (using the same ento-epidemiological parameters) the calibration takes care of climate-related model biases only.

**Figure 3:**
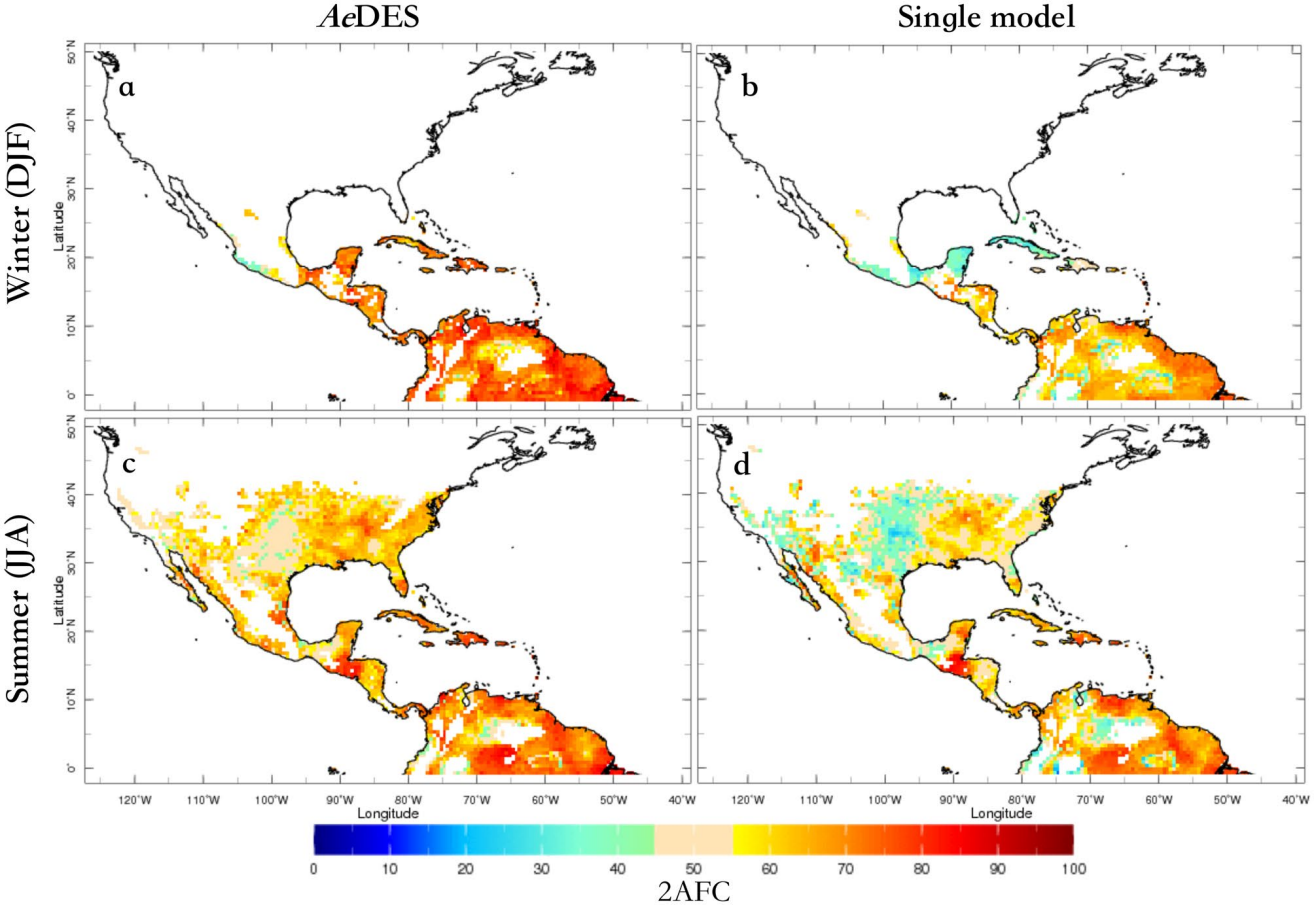
Cross-validated skill assessment (using 2AFC) between the *Ae*DES multi-${\mathscr {R}}_0$ model system (**a**,**c**) and the Caminade et al.^21^ ${\mathscr {R}}_0$ model, for the boreal winter (**a**,**b**; DJF: Dec–Feb) and summer (**c**,**d**; JJA: Jun–Aug) seasons. Values above (below) 50% indicate better (worse) discrimination than long-term averages; only values corresponding to suitable conditions for transmission (${\mathscr {R}}_0>1$) are plotted. Skill of the other involved models is similar to the Caminade et al.^21^ one (see Data and Codes Availability).

To evaluate how good the ${\mathscr {R}}_0$ forecasts are, a skill assessment is conducted. Because direct measurements of ${\mathscr {R}}_0$ with which to compare model predictions are not available, the predictive skill of the *Ae*DES forecast sub-system is assessed comparing its predictions against the output of the monitoring sub-system. Specifically, observed climate (temperature) values are used to calculate the “observed” ${\mathscr {R}}_0$, which is used as benchmark or reference in the skill assessment. Climate hindcasts (i.e., retrospective climate forecasts) are used to force the ${\mathscr {R}}_0$ models, thus producing the ${\mathscr {R}}_0$ forecasts (see Fig. 1). The cross-validated skill assessment compares the “observed” and forecast ${\mathscr {R}}_0$ values, and helps analyze how biases in climate hindcasts carry through to influence ${\mathscr {R}}_0$.

The skill assessment was conducted for each calibrated ${\mathscr {R}}_0$ model and the final multi-${\mathscr {R}}_0$ model ensemble (i.e., the *Ae*DES model), focusing on discrimination as an actual measure of the value of a forecast sub-system^31^. Although correlations between forecasts and observation are often used to assess skill, they only provide information of how in phase or not the forecasts are with respect to observations. The metric selected for skill assessment was the two-alternative forced choice, or 2AFC, which “measures the proportion of all available pairs of observations of differing category whose probability forecasts are discriminated in the correct direction”^31^. In other words, when terciles (above-normal, normal, below-normal conditions) are used, the 2AFC measures how well the system distinguishes between the different categories; a system with poor discrimination is of no practical and economical value for decision makers. Furthermore, 2AFC has an intuitive interpretation as an indication of how often the forecasts are correct^31^.

*Ae*DES’s predictive skill (as measured by 2AFC) is well above that of the reference long-term average (corresponding to 2AFC = $50\%$), with values $\sim$1.4–1.8 times larger than that baseline basically everywhere in the region under study. Skillful regions extend farther north during the boreal summer (Jun–Aug, or JJA) due to more suitable areas for the vectors because of higher seasonal temperatures (see Fig. 3a,c). Also, as expected, *Ae*DES exhibits skill improvement compared to any of the models involved in its ensemble (Fig. 3), which show comparable skill distributions among themselves, both in space and time. *Ae*DES tends to outperform the individual models everywhere, but especially in the Caribbean (e.g., Cuba, Jamaica, Haiti, and Dominican Republic) and in a lower degree in the United States Great Plains, southern Mexico, Colombia’s Orinoquía and the northern Amazon in Brazil (Fig. 3); it also outperforms its predecessor model for Latin America and the Caribbean, described by Muñoz et al.^8^, especially in summer in western Colombia, and in winter in most of Central America and the Yucatan Peninsula (cfr. Fig. 4 in Muñoz et al.^8^).

Predictive skill of the *Ae*DES system is especially high (2AFC $\sim$70–90%) in most locations of Central America, the Caribbean and northern South America in boreal winter (Dec–Feb, or DJF), with “skill hotspots” in both boreal summer and winter in Guatemala, Honduras, El Salvador, Cuba, Haiti and Dominican Republic, Jamaica, Puerto Rico, and some island nations in the Lesser Antilles (unfortunately the observational dataset used for calibration does not cover all of these island nations).

Regarding North America, the Yucatan Peninsula is one of the locations with highest skill, especially in DJF, a peak season for tourism, and thus increased human mobility. In summer, almost the entire Pacific coast of Mexico exhibits 2AFC values above 65%. Overall, predictive skill over the United States in summer tends to be higher in the eastern half of the country than in the western half (where orographic temperatures naturally tends to control vector proliferation in large regions), and ranges between 50 and 90% along the United States-Mexico border and the states along the Gulf of Mexico’s shoreline. Forecast discrimination skill for southern Florida is also high in summer (values $\sim$ 90%, see Fig. 3c). In northern South America, the Caribbean coast of Colombia, and northern regions of Venezuela, Guyana, Suriname, French Guyana, and northeastern Brazil exhibit very high skill both in summer and winter.

Hence, predictive discrimination skill of *Ae*DES is in general high, and decision makers geographically interested in the hotspots mentioned above can take special advantage of the enhanced skill of the system in these regions to improve their response times on key prevention and control strategies, at least one month ahead of the target season, in locations known to have the vector(s) and the pathogen(s). Conversely, in other places decision makers could use the information provided by the *Ae*DES system on suitable environmental conditions to show how important it is to prevent the vectors from establishing in new geographical locations.

## Discussion

The risk of *Aedes*-borne disease transmission is in general very difficult to estimate, in part due to the complexities of accurately assessing the actual risk in terms of hazards and vulnerabilities impacting the target population. The general approach should successfully integrate the interactions between humans, virus, vectors, and the environment, making it a very complex system to forecast, in particular because many of those interactions are not yet well understood. An alternative is to identify a predictand (variable to monitor and predict) that (a) enables decision makers to take timely, “no-regrets” actions, (b) is verifiable (can be easily obtained from the information available or the health surveillance systems in place), and (c) can be skillfully predicted for the region and timescales of interest. The information provided to decision makers does not need to be perfect, but it needs to be reliable enough to improve decision-making.

Typical choices of predictands in the case of interest are number of positive cases and incidence. Although these options generally satisfy criteria (a) and (b) mentioned above, predictive skill tends to be a barrier to making the best decisions in a *timely* manner. Low predictive capacity for these predictands occurs for different reasons, but often can be traced to the fact that they depend on a variety of complex factors (e.g., socio-economic conditions, human behavior, human mobility, etc.), some of which are (still) largely unpredictable. Previous work has argued^8^ that a potential alternative is to focus on environmental suitability for transmission, since variables like temperature, relative humidity, vegetation cover and sometimes rainfall, are skillfully predictable at timescales decision makers are interested in. In this sense, climate imparts predictability to the *Aedes*-borne diseases transmission problem if a predictand like temperature-dependent ${\mathscr {R}}_0$ is used as a proxy for environmental suitability (and, under certain conditions, transmission risk), even when clearly it is not representing the complete risk picture: additional information, as mentioned earlier, on the presence of the vector(s), the population exposed to the disease, and circulation of the virus is also needed. Recent work by Monaghan et al.^32^ uses a similar approach to the one presented here to address the vector presence/absence component of the problem, and certainly both systems could be combined to provide additional information for decision makers in the health sector.

### *Ae*DES, uncertainties and decision-making

**Figure 4:**
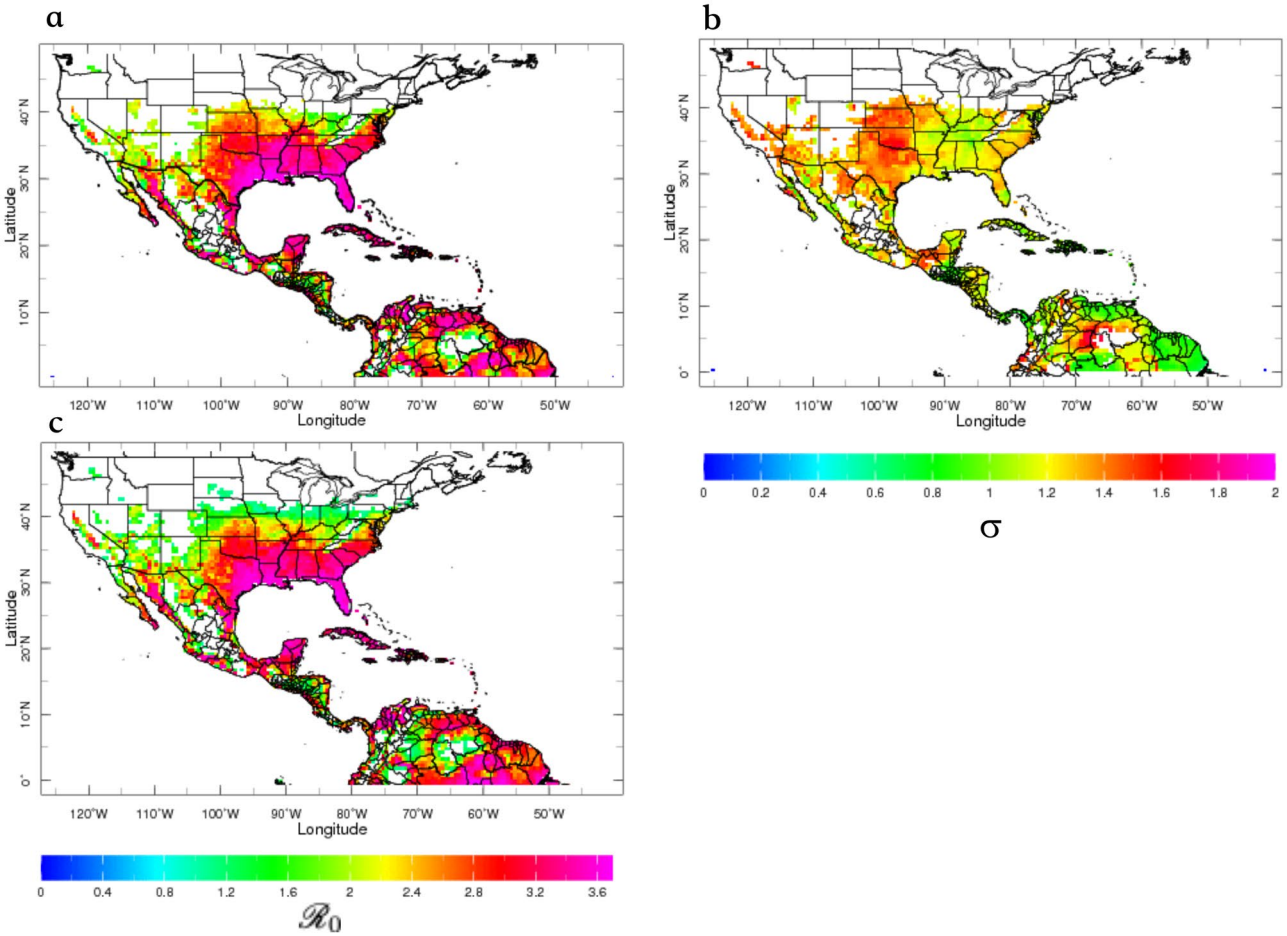
Example of deterministic forecasts for Jun–Aug 2016, initialized in May 2016. (**a**) AeDES seasonal forecast of the expected value of ${\mathscr {R}}_0$, along with the (**b**) forecast standard deviation ($\sigma$), presented to provide decision makers information about the forecast uncertainty. (**c**) Actual seasonal values of ${\mathscr {R}}_0$, provided by the AeDES monitor sub-system.

**Figure 5:**
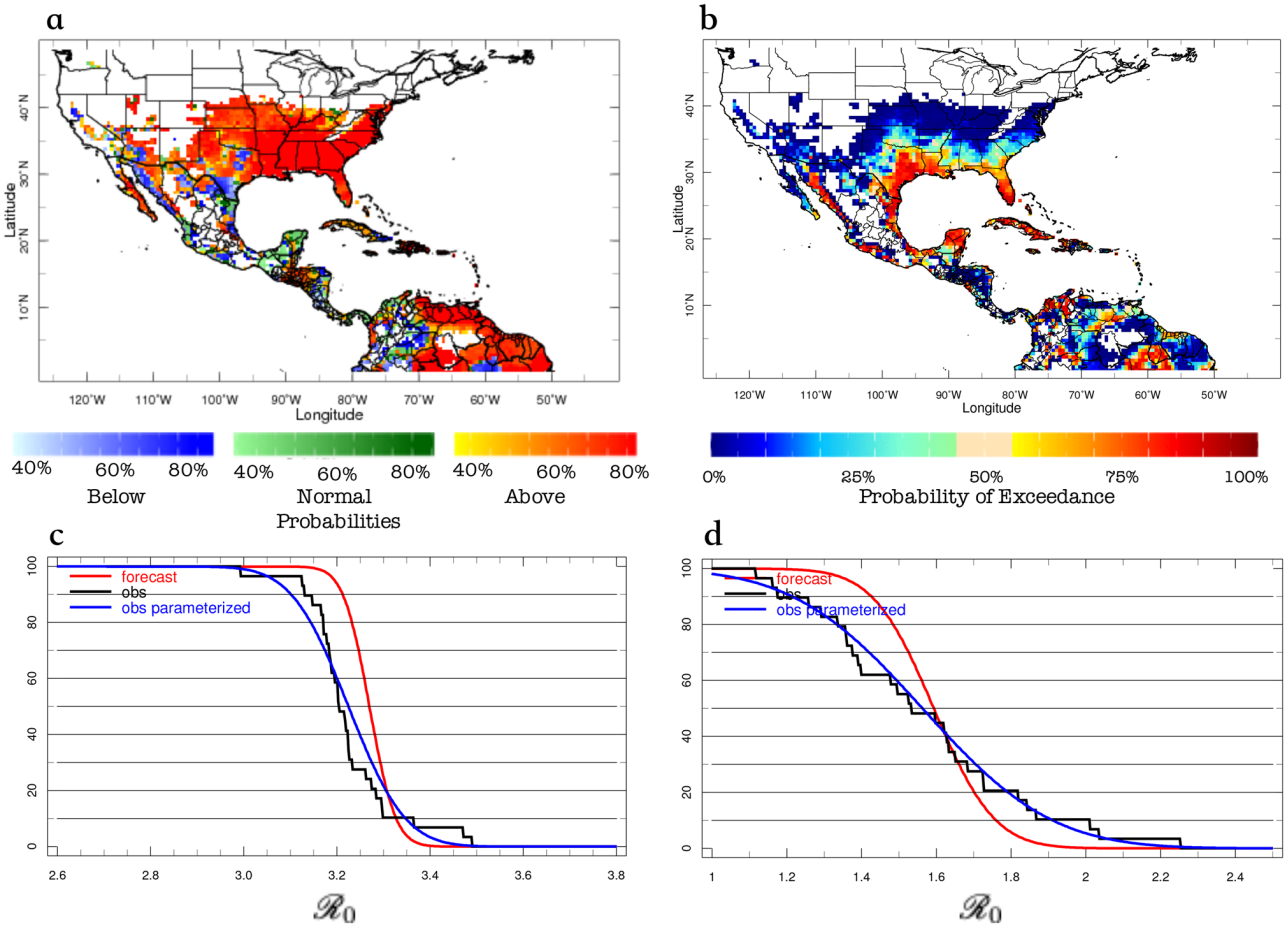
As in Fig. 4 but for probabilistic forecasts. (**a**) Tercile-based forecast; with uncertainty presented in terms of probabilities for each category: below-normal (in blue), normal (green) and above-normal (in red). (**b**) Spatial forecast of probabilities of exceeding ${\mathscr {R}}_0 = 3$. In the bottom panels, the “observed” (empirical in black, smoothed/parameterized in blue) and forecast (red) probability of exceedance distributions for (**c**) Miami, FL, and (**d**) New York City, NY, are also presented for comparison.

A large amount of work in the related scientific literature has been focused on developing or improving different ${\mathscr {R}}_0$ models (see Van den Driessche^33^ and references therein), but few efforts have addressed real-time, objective ${\mathscr {R}}_0$ seasonal forecasts, and no such operational system—to the best of our knowledge—existed until now for *Aedes* spp. in North America, Central America, northern South America and the Caribbean basin. Furthermore, to better assess uncertainty in *Ae*DES, the approach followed here involves the use of not one but multiple ento-epidemiological models, forced by state-of-the-art seasonal climate models from the National Oceanic and Atmospheric Administration (NOAA) North American Multi-Model Ensemble project^34^.

There is consensus in the scientific community on the need to include uncertainty information on any forecast that is produced^35^. One way of providing that information is to add confidence limits if the forecasts are deterministic (calculated values of ${\mathscr {R}}_0$ in our case). To give an example, Fig. 4 illustrates the expected ${\mathscr {R}}_0$ values for the summer of 2016 (Fig. 4a), and the expected standard deviation (or uncertainty, Fig. 4b); for reference, the monitored (or “observed”) values for the same summer are presented in Fig. 4c).

Another way to provide information about the forecast uncertainty is via the use of probabilities to indicate how confident (or not) the system is that a certain outcome—say, above normal ${\mathscr {R}}_0$ values—will occur during the next season. An example of a tercile-based ${\mathscr {R}}_0$ probabilistic seasonal prediction, again for the summer of 2016, is presented in Fig. 5a, where probabilities of below-normal, normal, or above-normal ${\mathscr {R}}_0$ values correspond to red, green, and blue color shades, respectively. Although this is generally very useful information, and tercile-based probabilistic forecasts have been used for more than two decades now, decision makers often require information beyond the usual three categories described above. Using the entire probability density function (see “Methods” section), *Ae*DES also provides probabilities of exceeding particular thresholds of interest (Fig. 5b).

**Figure 6:**
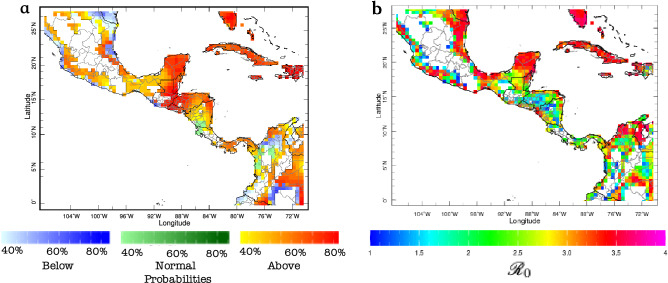
Real-time environmental suitability forecasts for Central America, for Jul–Sep 2020, initialized in June 2020. (**a**) Tercile-based probabilistic forecast; with uncertainty presented in terms of probabilities for each category: below-normal (in blue), normal (green) and above-normal (in red). (**b**) Deterministic forecast.

To illustrate the use of both deterministic and probabilistic forecasts, we analyze a hindcast for 2016 (Figs. 4, 5, and a real-time forecast for 2020 (Fig. 6). Consider first the recent Zika epidemic in the Americas^36^, which in the US led to $1.1 billion^37^ of public spending for emergency response. Official CDC numbers^38^ for Zika cases in the US indicate that both Miami, FL, and New York City (NYC), NY, reported slightly more than 1,000 cases in 2016, around 40% of the total number of cases in the US. Most of these cases were reported after the summer of 2016, a period of increased environmental suitability and human mobility (e.g., tourism to the Caribbean). We will focus on these two cities in this example.

By the beginning of May 2016, decision makers using *Ae*DES would have expected enhanced suitability conditions for Zika during JJA in basically all of the southeastern US states, but also the Caribbean, most of Central America and northern South America (Figs. 4a,b, 5a), where several Zika cases had been already reported. Although it was highly probable that both Miami and NYC exhibited above-normal suitability conditions (Fig. 5a), only Miami was expected to exceed ${\mathscr {R}}_0=3$ (Fig. 5b). In fact, the decision makers could have used *Ae*DES to determine that most probably Miami would not exceed ${\mathscr {R}}_0=3.4$ (Fig. 5c), while NYC most probably would not exceed ${\mathscr {R}}_0=2$ (Fig. 4d). These probabilistic forecasts were consistent with the deterministic ones for both cities (Fig. 4a,b), and by early September 2016—once the actual summer ${\mathscr {R}}_0$ values were available in the monitor sub-system—decision makers would have discovered that the forecasts were actually correct (Fig. 4c). But coming back to May 2016, what do those particular ${\mathscr {R}}_0$ forecasts mean? It depends on whether the location of interest already has the virus and the vector present, and a susceptible, exposed, and unprepared population. If the virus and the vector(s) are not present, the ${\mathscr {R}}_0$ maps just indicate that the environment is suitable for transmission, but there is no immediate risk. In some cases, preventive measures can be taken to avoid the future presence of the virus and the vector.

If all the conditions of vector and virus presence and population susceptibility and exposure are met, then given an original number of, for example, 40 Zika cases and a generation time of 20 days (15.6–25.6 days; standard deviation of 7.4 days)^39^, an ${\mathscr {R}}_0=2$ means that after four generations—each spaced $\sim 20$ days—or in about 3 months, there would be a total of 600 locally transmitted cases arising from the original 40. Since ${\mathscr {R}}_0$ is proportional to the duration of infectivity, an ideal action would be to reduce the infective period of cases, such that the effective reproduction number, ${\mathscr {R}}$, is reduced. For example, in NYC, with an expected ${\mathscr {R}}_0<2$ for JJA 2016, any combination of strategies to reduce the effective duration of infectivity by over 50% would mean an average ${\mathscr {R}}<1$, which should stop the spread of Zika over time. Beyond the obvious vector control strategies (for which knowing in advance when it is not going to rain could be useful), increasing traveler health surveillance, reducing the symptom-onset-to-isolation times, and the mosquito bite rates via specialized clothing and personal protective items can all help decrease the reproduction number. Economic costs for fighting the Zika epidemic would be most probably higher for Miami, given the higher ${\mathscr {R}}_0$ value forecast for the summer.

As our second example, consider now the current dengue outbreak in Central America. To provide context, the number of dengue cases reported in 2019 was the highest on record in the Americas^40^, and 30% larger than those reported in 2015. By the end of July 2019, Honduras, Guatemala, and Nicaragua had declared an epidemiological alert, and cases kept increasing in those and other countries in Central America during the rest of the year, and during 2020^40^. Four dengue serotypes (DENV1, DENV2, DENV3, and DENV4) are known to be presently circulating in the region.

The *Ae*DES real-time forecast, initialized in June 2020, indicates relatively high probabilities of above-normal environmental suitability for most of Central America for July–September 2020, with most locations exceeding ${\mathscr {R}}_0$ = 2 (Fig. 6). The most recent (May 2020) seasonal climate forecasts provided by the International Research Institute for Climate and Society (IRI)^41^ suggest above-normal temperatures during the summer for most of the Yucatan peninsula, Guatemala and Honduras, and above-normal rainfall for almost all of Central America for that same season. Furthermore, the IRI forecasts also indicate increased probabilities for a borderline La Niña during the second half of 2020, which is typically (but not always) associated with above-normal rainfall conditions in the region. Together, all these predictions suggest the present dengue outbreak will continue during the rest of 2020 and most likely will worsen.

The situation is exacerbated by the fact that SARS-CoV-2 has increased the population vulnerability in Central America by, among others, increasing food insecurity, which can impact the immune system of the average individual (increased susceptibility); decreasing human mobility (quarantine in households without air conditioning and poor environmental conditions can increase exposure to mosquitoes^15,26^); and disrupting or hampering access to health services (decreased adaptive capacity). Furthermore, this compound effect involving the dengue and SARS-CoV-2 outbreaks is also expected to increase the number of coinfections in the region^42^.

Concrete actions to deal with the situation are always context-specific and need to be coordinated with the local authorities. *Ae*DES can help providing information to make those decisions; for example, rather than assigning resources homogeneously to all regions in a country, or assuming that the present distribution of resources will be the same in the next following months, *Ae*DES can be used to help decide which regions require more or less attention during the next season depending on specific thresholds and triggers, and how the resources need to change also in time. A combination of environmental suitability forecasts with weather and climate forecasts, can also inform when to implement particular actions within the season; for example, extreme rainfall (e.g., during a potential La Niña in the coming months) can hamper vector control activities, provision of health services and medicines, transportation of goods and people, etc., and knowing with a high level of certainty when and where to act can be hugely beneficial to society.

### A NextGen climate-and-health service for multiple timescales

*Ae*DES is a “next generation” system because (1) it successfully tailors global climate information to be used at regional scales, (2) pattern-based calibration targeting mean, amplitude, and spatial biases is performed using a monitoring system based on the same variable that is being predicted, and (3) it produces tailored deterministic and probabilistic forecasts for user-selected thresholds of interest, including the use of the entire probability density function (also known as “forecasts in flexible format”^35^), to better assess uncertainties.

Previous research^8,11,16^ has underscored the importance of analyzing climate signals at multiple timescales to improve decision-making processes in the health sector. In particular, Muñoz et al.^16^ and Thomson et al.^11^ have shown that the seasonal-to-interannual timescale tends to explain most of the total variance observed in climate variables impacting vector-borne disease transmission, like temperature and rainfall. Hence, although the long-term climate change and natural decadal variability signals also are considered, *Ae*DES pays special attention to continuously providing actionable information at seasonal-to-interannual timescales, which along with the weather and sub-seasonal^43^ scales are the most often used for health early warning systems.

Due to large uncertainties in long-term climate projections, the present approach should in general not be used in combination with climate change scenarios. Nonetheless, the same approach is adequate for shorter-term timescales, like the sub-seasonal (roughly 2–6 weeks^43^) or weather (0–2 weeks) ones. Providing actionable information at multiple timescales (e.g., via the IRI’s Ready-Set-Go approach^35^). The team is presently exploring when and where predictive skill at these timescales is high enough to guide decision-making processes in the health sector, taking advantage of windows of opportunities in forecasts at those timescales^44^.

## Methods

### Data

All analyses are conducted for the geographical domain defined by the coordinates 126° W–40° W and 1° S–50° N (Fig. 1). The reference or “normal” period corresponds to 1982–2010.

Rather than focusing on particular diseases, here we considered common environmental thresholds and ento-epidemiological parameters for *Aedes*-borne diseases as a whole. If the parameters are well known for diseases of interests, then the same approach can be used to have tailored information for those cases. For consistency with previous studies and model validations, we used the same ento-epidemiological parameters reported by Liu-Helmersson et al.^23^, Wesolowski et al.^22^, Caminade et al.^21^ and Mordecai et al.^24^. The equations and parameter choice can be found in those references, and in our scripts used to build *Ae*DES (see Data and Codes Availability below).

Two types of near-surface (2 meter) temperature datasets were used: observations and forecasts. Observations consist of gridded fields from NCEP’s GHCN-CAMS^25^ project, at 0.5 degree resolution. When designing the monitoring sub-system, additional observation datasets were used to compare between different spatial resolutions; these products are CRUv4^45^, at 0.5 degree resolution, and PRISM^46^, at 0.042 degrees. All products were interpolated to the lower resolution (0.5 degree), and spatial correlations were computed for the 1982–2010 period. No statistically significant differences were observed between the products at p = 0.05, using a simple t-Student test (not shown).

Given that the GHCN-CAMS dataset is freely updated every month and covers not only North America but also the boundary regions of interest (the Caribbean, Central America and northern South America), this product was selected to force the multi-${\mathscr {R}}_0$ model for the monitoring sub-system, in the same way the *Ae*DES hindcasts (described below) were computed, except that only one “realization” (the observed climate) was used. Although ${\mathscr {R}}_0$ is not a direct observable, for simplicity in this paper we refer to this set of environmental suitability maps as the “observed” (or actual) ${\mathscr {R}}_0$. The approach is general and does not require GHCN-CAMS to work; if a different reliable observed temperature dataset is available (for example at higher spatial resolution), the system can use it. We recognize different products can exhibit important biases with respect to local station data, for example in relatively small and topographically complex island nations in the Caribbean. The *Ae*DES system can be implemented locally in those cases to use a gauge-based dataset; in some cases, systematic biases between such dataset and GHCN-CAMS can be identified so the present system can still be used after local calibration.

The other temperature dataset consists of seasonal predictions from all models operationally available in the North American Multi-Model Ensemble (NMME) project^34^, as in Muñoz et al.^8^, consisting of a total of 96 ensemble members. *Ae*DES presently uses the latest version of the Canadian climate model (CanSIPv2), after the older Canadian models were discontinued in August 2019.

In addition, the monitoring sub-system also presents infant mortality data from the Socioeconomic Data and Applications Center (SEDAC; https://sedac.ciesin.columbia.edu/data/collection/povmap), and the Gridded Population of the World, Version 4 (GPWv4), Revision 11^47,48^.

### ${\mathscr {R}}_0$ models and design of the next generation forecast sub-system

We used four previously developed ${\mathscr {R}}_0$ models for *Aedes*-borne diseases, described in detail by Caminade et al.^21^, Liu-Helmersson et al.^23^, Wesolowski et al.^22^ and Mordecai et al.^24^. These ${\mathscr {R}}_0$ models include terms for several temperature-dependent processes that affect mosquito-borne transmission, including: mosquito biting rate; vector competence, or the proportion of infectious bites that infect susceptible humans and the proportion of bites on infected humans that result in mosquito infection; adult mosquito mortality rate; extrinsic incubation period (EIP), or the time between an uninfected mosquito biting an infected human and the mosquito becoming infectious; the daily egg production rate by a female mosquitoes, and egg- and juvenile-stage survival probability and development rates.

Results from Mordecai et al. show a unimodal response to temperature for dengue transmission, with transmission by *Ae. aegypti* peaking at 29.1 °C (95% CI 28.4–29.8 °C), but occurring between 17.8 and 34.6 °C^24^. Transmission by *Ae. albopictus* was shown to peak at 26.4 °C (95% CI 25.2–27.4 °C), but occurred between 16.2 and 31.6 °C. Biological traits such as adult survival, vector competence, and extrinsic incubation period vary across vector species and upon infection with different arboviruses^49^. However, modeling studies of Zika and dengue transmission by *Ae.* *aegypti* show that optimal temperatures for vector competence and EIP are similar for both viruses. For example, vector competence for dengue peaked between 31 and 32 °C in both mosquito species, and for Zika peaked at 30.6 °C in *Ae. aegypti*^24,50^. Meanwhile, the EIP optimum for dengue was approximately 35 °C for both mosquito species, and for Zika was 36.4 °C in *Ae. aegypti*. Therefore, at regional and continental scales, we find sufficient evidence to discuss ${\mathscr {R}}_0$ values as parameterized for *Aedes*-borne diseases as a whole. Mathematically, the contribution to the total ${\mathscr {R}}_0$ presented in *Ae*DES of each *Aedes* vector is computed, as usual, via the Euclidean norm (e.g., see Eq. (19) in Muñoz et al.^8^, or Eq. (1) and supplemental material in Caminade et al.^21^).

To build the ${\mathscr {R}}_0$ hindcasts, all four ${\mathscr {R}}_0$ models were forced independently using each one of the 96 NMME climate realizations (i.e., a total of 384 realizations) in hindcast—or retrospective forecast—mode for each season and year in the 1982–2010 period (29 years). For example, all May initializations in the 1982–2010 period were used to obtain the 29 NMME temperature hindcasts JJA seasons, which were then used to force each one of the ${\mathscr {R}}_0$ models to produce the 1982-2010 environmental suitability hindcasts. Hence, a total of 4,608 (4 ${\mathscr {R}}_0$ models times 12 initializations times 96 climate model members) 29-year long seasonal hindcasts were produced.

We then performed a pattern-based calibration for each season and model independently, to avoid mixing models with different characteristics when correcting for mean, amplitude, and spatial biases. Following the approach of Muñoz et al.^8^, the calibration method selected was principal component regression (PCR)^51^. The PCR-based calibration approach builds a regression model for each grid box of the “observed” ${\mathscr {R}}_0$ field, using a linear combination of the hindcast ${\mathscr {R}}_0$’s Empirical Orthogonal Functions (EOFs). These EOFs represent spatio-temporal patterns of ${\mathscr {R}}_0$ in each model, and vary in magnitude, space, and location through the initializations—one per calendar month. For a given initialization, say January, and set of EOFs, each grid box has in general a different set of regression coefficients associated with each one of the EOF. The best cross-validated models were identified using a leave-five-out cross-validation window and the value of Kendall’s $\tau$ coefficient, which also defines the final number of EOFs for each model. Since a different PCR model is built for each grid box in the predictand domain, the number of EOFs can vary not only depending on the season. To avoid overfitting, we required a maximum of 5 EOFs, so the number ranged from 2 to 5 EOFs, depending on the season.

The *Ae*DES multi-${\mathscr {R}}_0$ calibrated ensemble mean was computed using each calibrated model, providing deterministic outcomes, and the entire probability density function (PDF) for ${\mathscr {R}}_0$, computing in that case the average of the Gaussian distribution parameters for each grid box (i.e., an ensemble built in the “probability space”). This means that, for each grid box, we independently averaged the location and shape parameters of the Gaussian distribution (i.e., the mean and the variance) provided by each one of the 384 members of the ensemble. Thus, each grid box has a final probability density function defined in terms of the average mean and the square-root of the average variance. We repeated this process for each of the 12 initializations. Note that the thresholds defining the below-normal and above-normal categories are computed independently for each grid box and season, using the mean and standard deviation defining the probability density functions produced by the monitor sub-system.

The PDF is used to offer uncertainty information for decision makers in the health sector and to compute the forecast probability of exceeding thresholds selected by the user. Tailored probabilistic forecasts produced using the entire PDF are often called “forecasts in flexible format”^35^. These products are available in the *Ae*DES Maproom (https://aedes.iri.columbia.edu).

The forecast skill assessment was conducted for each calibrated ${\mathscr {R}}_0$ model and the *Ae*DES ensemble system for the 1982–2010 period, using the “actual” ${\mathscr {R}}_0$ (computed from the observed climate data) available from the monitoring sub-system as reference, and the two-alternative forced choice^31^ (2AFC) metric. As indicated in the “Forecast sub-system calibration and evaluation” section, the 2AFC measures discrimination, or how well, for example, the system can distinguish between above-normal, normal, and below-normal categories. For a given location, values of 100% indicate *Ae*DES has perfect discrimination, and values below 50% indicate that the system has worse discrimination than considering the 29-year-long average for that particular location, and thus are values associated with no predictive skill. Two contrasting seasons were selected for analysis in this study: boreal winter and summer.

The calibration and skill assessment processes were computed using the International Research Institute for Climate and Society’s (IRI) Climate Predictability Tool (CPT) version 16.3.2^52^, and its Python interface^53^ (PyCPTv1.7; https://bitbucket.org/py-iri/iri-pycpt/src/master/) to facilitate the mass production of the different hindcasts, skill assessment maps and forecasts. The resulting files were migrated to the IRI Data Library for public archiving and plotting.

## Data Availability

All input and produced data is freely available at the International Research Institute for Climate and Society’s Data Library: http://iridl.ldeo.columbia.edu/home/.agmunoz/.Aedes/#info.

## Code availability

Code is available on Muñoz’s GitHub account: https://github.com/agmunozs/Vectorbornediseases.
